# Unveiling the Role of TMPRSS2 in the Proteolytic Activation of Pandemic and Zoonotic Influenza Viruses and Coronaviruses in Human Airway Cells

**DOI:** 10.3390/v16111798

**Published:** 2024-11-20

**Authors:** Marie Schwerdtner, Luna C. Schmacke, Julia Nave, Hannah Limburg, Torsten Steinmetzer, David A. Stein, Hong M. Moulton, Eva Böttcher-Friebertshäuser

**Affiliations:** 1 Institute of Virology, Philipps-University Marburg, 35043 Marburg, Germany; marie.schwerdtner@uni-marburg.de (M.S.);; 2 Institute of Pharmaceutical Chemistry, Philipps-University Marburg, 35037 Marburg, Germany; 3 Department of Biomedical Sciences, Carlson College of Veterinary Medicine, Oregon State University, Corvallis, OR 97331, USA

**Keywords:** influenza A virus, coronavirus, TMPRSS2, proteolytic activation, SARS-CoV, MERS-CoV

## Abstract

The zoonotic transmission of influenza A viruses (IAVs) and coronaviruses (CoVs) may result in severe disease. Cleavage of the surface glycoproteins hemagglutinin (HA) and spike protein (S), respectively, is essential for viral infectivity. The transmembrane serine protease 2 (TMPRSS2) is crucial for cleaving IAV HAs containing monobasic cleavage sites and severe acute respiratory syndrome (SARS)-CoV-2 S in human airway cells. Here, we analysed and compared the TMPRSS2-dependency of SARS-CoV, Middle East respiratory syndrome (MERS)-CoV, the 1918 pandemic H1N1 IAV and IAV H12, H13 and H17 subtypes in human airway cells. We used the peptide-conjugated morpholino oligomer (PPMO) T-ex5 to knockdown the expression of active TMPRSS2 and determine the impact on virus activation and replication in Calu-3 cells. The activation of H1N1/1918 and H13 relied on TMPRSS2, whereas recombinant IAVs carrying H12 or H17 were not affected by TMPRSS2 knockdown. MERS-CoV replication was strongly suppressed in T-ex5 treated cells, while SARS-CoV was less dependent on TMPRSS2. Our data underline the importance of TMPRSS2 for certain (potentially) pandemic respiratory viruses, including H1N1/1918 and MERS-CoV, in human airways, further suggesting a promising drug target. However, our findings also highlight that IAVs and CoVs differ in TMPRSS2 dependency and that other proteases are involved in virus activation.

## 1. Introduction

Influenza viruses and coronaviruses (CoVs) are enveloped viruses causing respiratory diseases that range from mild to severe in humans. They belong to the *Orthomyxoviridae* and *Coronaviridae* families, respectively. Influenza A viruses (IAVs) are categorized into 19 distinct hemagglutinin (HA) and 11 neuraminidase (NA) subtypes based on the antigenic characteristics of their envelope proteins. Wild waterfowl serve as the natural host reservoir for IAVs [1]. Human IAV subtypes H1N1 and H3N2, along with influenza B virus (IBV), are responsible for seasonal flu epidemics, resulting in 3–5 million cases and 290,000–650,000 deaths annually [2]. Additionally, IAV pandemics can occur when new viruses emerge from avian IAVs and cross species barriers. The H1N1 pandemic in 1918 that caused an estimated 50 million deaths worldwide has been the most devastating one to date [3].

CoVs comprise a wide variety of viruses infecting a broad spectrum of vertebrate species. Seven human-pathogenic CoVs have been identified, including the four low-pathogenic human CoVs (HCoVs) that cause 15–30% of seasonal common colds [4]. Since the early 21st century, three additional CoVs have emerged through zoonotic transmission, causing severe respiratory disease in humans. The severe acute respiratory syndrome (SARS)-CoV appeared in 2002, resulting in around 8000 cases with a case fatality rate of ~10% [5,6]. In 2012, the Middle East respiratory syndrome (MERS)-CoV emerged, causing sporadic outbreaks with a ~35% case fatality rate [7,8]. While MERS-CoV transmission is considered to be primarily zoonotic, from dromedary camels to humans, limited human-to-human transmission among close contacts and in health care settings has been reported [9,10,11,12]. Recently, SARS-CoV-2, causing CoV disease 2019 (COVID-19), spread globally, resulting in over 776 million reported cases and more than 7 million deaths as of 19th September 2024.

**Figure 1:**
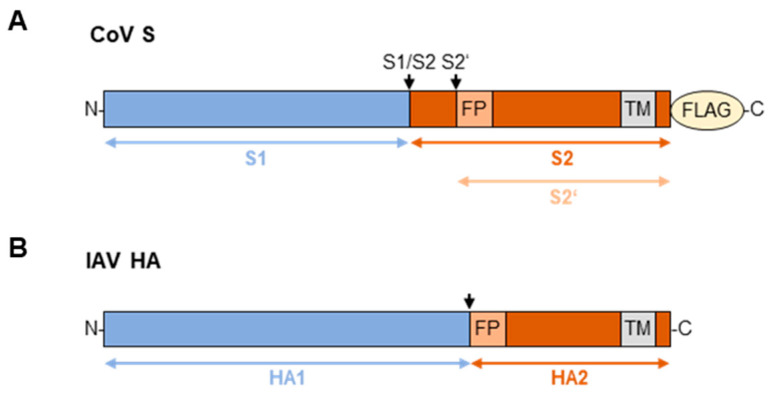
**Schematic representation of CoV S and IAV HA.** CoV S (**A**) and IAV HA (**B**) are synthesized as the inactive precursor proteins S0 and HA0, respectively, which require proteolytic processing for activation. Cleavage sites are indicated by arrows. Recombinant S was expressed with a C-terminal FLAG-tag in this study. FP = fusion peptide, TM = transmembrane domain.

Both IAV and CoV virions display class I viral fusion proteins on their surface, the HA and the spike protein (S), respectively, that facilitate receptor binding and membrane fusion. HA and S are synthesized as precursor proteins and require proteolytic activation by cellular proteases to gain fusion capacity (Figure 1) [13]. The precursor HA0 of IAV is cleaved post-translationally into its subunits HA1 and HA2 at a single cleavage site adjacent to the fusion peptide [14]. This cleavage is essential for a low pH-dependent conformational change in the protein that allows for insertion of the fusion peptide into the host endosomal membrane, initiating the formation of a fusion pore and ultimately facilitating the release of the viral RNA genome into the target cell [15]. In contrast, CoV S requires cleavage at two distinct sites, designated as S1/S2 and S2′ [16]. The cleavage at S1/S2 separates the S1 and S2 subunits and is generally assumed to occur after protein synthesis in the virus-producing cell, although a complete understanding of this cleavage event and the functions of its products is still under development. The S1/S2 cleavage event might be necessary for the conformational changes required for receptor binding or for the subsequent S2′ site cleavage by cellular proteases [17,18]. Once-cleaved S is incorporated into virions and is only processed at the S2′ site after receptor binding at or near the plasma membrane of the new target cell. This cleavage leads to the exposure of the fusion peptide, enabling membrane fusion and the release of the viral genome into the host cell [19,20].

Avian IAVs are classified into highly pathogenic (HPAIVs) and low pathogenic avian influenza viruses (LPAIVs) based on the level of their pathogenicity to poultry. Further, HPAIVs and LPAIVs can be distinguished by their HA cleavage site motifs [14,21]. HPAIV HAs possess a multibasic cleavage site (R-X-R/K-R↓), processed by the ubiquitously expressed subtilisin-like protease furin and related proprotein convertases (PC), thereby contributing to systemic spread and high virulence. In contrast, LPAIVs and all human IAV subtypes are cleaved at a monobasic cleavage site (R↓) by trypsin-like proteases [21]. The type II transmembrane serine protease (TTSP) transmembrane protease serine S2 (TMPRSS2) has been identified as the major HA-activating protease for the human IAV subtypes H1N1, H3N2 and H7N9 in human airway cells, and for IBVs in human lung cells [22,23]. Moreover, TMPRSS2 was found to be crucial for the activation of the LPAIV HA subtypes H1-11 and H14-15 in human airway cells, with H16 showing an intermediate phenotype [24]. In vivo studies in mice confirmed that TMPRSS2 is essential for the replication and spread of H1N1, H7N9 and H2 within the respiratory tract, as TMPRSS2-knockout mice were protected from disease [25,26,27,28].

Beyond its well-studied role in IAV HA activation, TMPRSS2 is involved in the cleavage and activation of several CoV S. We and others demonstrated that the SARS-CoV-2 S1/S2 site is cleaved by furin during S synthesis, whereas the S2′ site is processed by TMPRSS2 during viral entry. Although endosomal cathepsins can facilitate S-mediated entry in the absence of TMPRSS2, furin and TMPRSS2 are indispensable for efficient viral replication in human airway cells [29,30,31,32]. Similar findings were observed with SARS-CoV S, where activation can occur after the cellular entry of virions via the endosomal pathway but can also be facilitated by TMPRSS2 during entry at or close to the cell surface [33,34,35,36]. Regarding the cleavage of MERS-CoV S S1/S2 and S2′ sites, studies have yielded conflicting results on whether one or both of the dibasic motifs are cleaved by furin and whether furin cleavage is required at all for viral replication [18,37,38,39,40,41]. Yet, TMPRSS2 has been recognized as an important activating protease for MERS-CoV S [42,43]. Interestingly, TMPRSS2 knockout mice exhibit reduced pathogenesis upon infection with SARS-CoV, MERS-CoV and SARS-CoV-2, emphasizing the protease’s pivotal role in CoV activation and pathogenesis [44,45,46].

TMPRSS2 presents a promising target for antiviral therapies against IAVs and CoVs. Moreover, glycoprotein cleavage by TMPRSS2 has been proposed to play a role in inter-species transmission and host adaptation [47]. Hence, in this study, we aimed to investigate and compare the dependency of highly pathogenic CoVs SARS-CoV and MERS-CoV, as well as the 1918 H1N1 pandemic IAV, on TMPRSS2 in human airway cells. We assessed the cleavage efficiency of CoV cleavage site motifs by recombinant TMPRSS2 using an enzyme kinetic assay. We then analysed the proteolytic processing and functional activation of SARS-CoV and MERS-CoV S upon co-expression with TMPRSS2, human airway tryptase (HAT), murine (m)TMPRSS4 and mTMPRSS13. To validate our findings in a more relevant setting, we infected Calu-3 human airway cells with authentic SARS- or MERS-CoV and investigated the role of TMPRSS2 during multicycle replication by knocking down TMPRSS2 expression using a peptide-conjugated phosphorodiamidate morpholino oligomer (PPMO), a delivery-enabled antisense oligomer. We also examined the proteolytic activation and multicycle replication of the recombinant 1918 H1N1 IAV in Calu-3 cells in the presence or absence of TMPRSS2. Finally, we explored the TMPRSS2 dependency of avian H12 and H13, as well as bat-derived H17, all of which could potentially cause zoonotic spillover to humans.

## 2. Materials and Methods

### 2.1. Cells

Calu-3 human airway epithelial cells (ATCC, HTB-55) were cultivated in DMEM/F-12 (Gibco, Waltham, MA, USA) supplemented with 10% fetal bovine serum (FBS), 1% glutamine and 1% penicillin/streptomycin. HeLa cervical epithelial cells (ATCC, CCL-2), HEK293 human embryonic kidney epithelial cells (ATCC, CRL-1573) and Madin–Darby canine kidney II (MDCKII) cells were grown in DMEM (Gibco, Waltham, MA, USA) supplemented with 10% FBS, 1% glutamine and 1% antibiotics.

### 2.2. Viruses

Infectious viral stocks of SARS-CoV (kindly provided by Verena Krähling, Institute of Virology, Marburg, Germany) and MERS-CoV (kindly provided by Lucie Sauerhering, Institute of Virology, Marburg, Germany) were generated by infecting Calu-3 cells at a low multiplicity of infection (MOI) and harvested by low-speed centrifugation of the supernatant. Recombinant (r)H1N1/1918 was generated using the eight-plasmid transfection system [48]. In brief, plasmids coding for all eight gene segments were co-transfected into HEK293 cells using Lipofectamine 2000 (ThermoFisher, Waltham, MA, USA, 11668019). After 48 h, the supernatant was treated with 1 µg/mL tosyl phenylalanyl chloromethyl ketone (TPCK)-treated trypsin (Sigma, St. Louis, MO, USA, T1426-1G) for 50 min at 37 °C to ensure HA activation, as HEK293 cells do not express an appropriate protease. The supernatant containing infectious virus was then used to inoculate Calu-3 cells. The virus stock was harvested at 72 h post infection (p.i.) by low-speed centrifugation of the supernatant and was further propagated on Calu-3 cells. All work with these viruses was conducted under BSL-4 conditions. Recombinant H12-, H13- and H17-SC35M were also generated using the eight-plasmid transfection system as described above, but rH12- and rH13-SC35M were propagated on MDCKII cells. The different HA-segments were combined with seven gene segments of A/seal/Massachusetts/1/80 (H7N7) (SC35M).

### 2.3. Plasmids

The expression vectors coding for SARS- or MERS-CoV S with a C-terminal FLAG-tag, pCMV3-MERS-Spike-cFLAG (VG40069-CF) and pCMV3-SARS-Spike-cFLAG (VG40150-CF), as well as a vector encoding human dipeptidyl peptidase 4 (DPP4) with a C-terminal HA-tag, pCMV3-hDPP4-cHA (HG10688-CY), were purchased from Sino Biological, Houston, TX, USA. The pcDNA3.1(+) plasmid encoding human angiotensin-converting enzyme 2 (ACE2) with a C-terminal HA-tag was commercially obtained from GeneART (ThermoFisher). The expression vector pCAGGS-H1/1918-HA codes for the HA of A/South Carolina/1/1918 (kindly provided by Mikhail Matrosovich). The expression vectors coding for human TMPRSS2 and HAT, pCAGGS-hTMPRSS2 and pCAGGS-HAT, respectively, were previously described [22]. The expression vectors coding for the murine proteases mTMPRSS4 and mTMPRSS13, pCMV6entry-mTMPRSS4-Myc-DDK (MR206946) and pCMV6entry-mTMPRSS13-Myc-DDK (MR220731), are described in [49]. The pHW2000 plasmids encoding the gene segments of A/seal/Massachusetts/1/80 (H7N7, SC35M) were kindly provided by Thorsten Wolff (Robert Koch Institute, Berlin, Germany). The cDNA encoding for the HAs of A/little yellow-shouldered bat/Guatemala/164/2010 (H17N10; GenBank accession number CY103884), A/northern shoveler/Interior Alaska/1/2007(H12N5; GenBank accession number CY038354) and A/black-headed gull/Netherlands/6/2007(H13N6; GenBank accession number MF575207) were purchased from ThermoFisher (Waltham, MA, USA) and cloned into the pHW2000 vector using BsmBI restriction sites. The pHW2000 plasmids carrying the genes of H1N1/1918 were kindly provided by Mikhail Matrosovich (Institute of Virology, Marburg, Germany). The HA sequence was based on A/South Carolina/1/1918 (H1N1) (GenBank accession number AF117241.1) and all other sequences were taken from A/Brevig Mission/1/1918 (H1N1; GenBank accession numbers PB2: DQ208309.1, PB1: DQ208310.1, PA: DQ208311.2, NP: AY744935.1, NA: AF250356.2, M: AY130766.1, NS: AF333238.1). Non-coding regions were added based on the closely related A/WSN/33 (H1N1).

### 2.4. Antibodies

The primary antibodies used for Western blot analyses were rabbit-anti FLAG (1:500; Sigma-Aldrich, St. Louis, MO, USA, F7425), rabbit-anti MERS-CoV-Spike-S2 (1:500; Sino Biological, Houston, TX, USA, 40070-T62), rabbit-anti SARS-CoV-2-Spike-S2 (1:500; ThermoFisher, Waltham, MA, USA, PA5-112048), rabbit-anti H1N1-HA (1:500; Sino Biological, Houston, TX, USA, 11085-T62), mouse-anti Tubulin (1:2000, Sigma-Aldrich, St. Louis, MO, USA, T9026) and mouse-anti β-actin (1:2500-5000; Abcam, Cambridge, UK, ab6276). The horse radish peroxidase (HRP)-conjugated secondary antibodies used for the detection of primary antibodies were goat-anti rabbit-HRP (1:6000; Agilent, Santa Clara, CA, USA, P0448) and rabbit-anti mouse-HRP (1:6000; Agilent, Santa Clara, CA, USA, P0260). The immunofluorescence staining of transiently expressed S was performed using mouse-anti FLAG M2 (1:50; Sigma-Aldrich, St. Louis, MO, USA, F1804) and goat-anti mouse-AlexaFluor^488^ (1:100; ThermoFisher, Waltham, MA, USA, A-10667). For the immune plaque assay, mouse-anti IAV-NP (1:5000; antibodies-online, ABIN349718) was the primary antibody, and the same HRP-conjugated secondary antibody was used as for Western blot analyses.

### 2.5. PPMO and Protease Inhibitors

The PMO T-ex5 (5′-AGAGTTGGAGCACTTGCTGCCC-3′) was synthesized at Gene Tools LLC (Corvallis, OR, USA). Subsequently, the peptide (RXR)_4_ (R = Arginine, X = 6-aminohexanoic acid) was covalently conjugated to the 3′ end of each PMO through a non-cleavable linker to produce peptide-PMO (PPMO) [50]. The furin-inhibitor MI-1851 (*K*i = 10.1 pM) was synthesized in-house as described previously in [51]. E64d, an inhibitor for endosomal cathepsins, was purchased from Selleckchem (Houston, TX, USA, S7393) and aprotinin was purchased from AppliChem (Darmstadt, Germany, A2132).

### 2.6. Multicycle Viral Replication in the Presence of Protease Inhibitors or PPMO

Calu-3 cells cultivated to confluency in 12-well plates were infected with SARS-CoV or MERS-CoV at an MOI of 0.001 for 1 h under serum-free conditions with DMEM supplemented with glutamine and penicillin/streptomycin (DMEM++). Subsequently, the cells were thoroughly washed and incubated in DMEM supplemented with 3% FBS, glutamine and penicillin/streptomycin (DMEM+++) with or without the addition of protease inhibitors or DMSO. Infection with rH1N1/1918 was performed accordingly but using an infection medium consisting of DMEM supplemented with 0.1% bovine serum albumin (BSA), glutamine and antibiotics for infection and incubation. The supernatant was sampled at indicated time points and subjected to tissue culture infectious dose 50% (TCID_50_) endpoint titration, as described below, to determine viral titers. For infection with rH12-, rH13- and rH17-SC35M, Calu-3 cells were cultivated to confluency in 24-well plates and inoculated at an MOI of 0.01–0.0001 in infection medium. After 1 h, the cells were washed and further incubated in infection medium. The viral titers in the supernatant were determined at indicated time points via an immune plaque assay, as previously described [52]. The PPMO treatment of Calu-3 cells was performed 24 h prior to infection by incubating the cells with or without 25 µM T-ex5 in infection medium. For the analysis of S or HA cleavage, Calu-3 cells were infected as described above but at a high MOI of 1. At 24 h p.i., the infected cells and supernatant were harvested and prepared for SDS-PAGE and Western blot, as described below.

### 2.7. Virus Titration by TCID_50_

Calu-3 cells were cultivated to confluency in 96-well plates before the medium was changed to DMEM+++ (SARS-CoV, MERS-CoV) or infection medium (rH1N1/1918). The supernatants taken from infected cells at indicated time points p.i. were serially diluted from 5^−1^ to 5^−11^ in four replicates directly on the cells. After 3–4 days, when a cytopathic effect (CPE) was clearly visible, the viral titers were calculated with the Spearman and Kaerber algorithm [53].

### 2.8. Transient Expression of Spike Protein in HeLa Cells and HA in HEK293 Cells

To analyse the cleavage of SARS-CoV or MERS-CoV S, HeLa cells were grown to near confluency in 12-well plates. The cells were co-transfected with 1.6 µg of expression plasmids coding for the cDNA sequence of SARS or MERS S, respectively, and 15 ng of expression plasmids coding for the cDNA of various trypsin-like serine proteases or an empty vector using Lipofectamine 2000 according to the manufacturer’s protocol. The analysis of rH1N1/1918 HA cleavage was performed by co-transfecting HEK293 cells grown to near confluency in 24-well plates with 0.8 µg of pCAGGS-H1N1/1918-HA and 7.5 ng of expression plasmids coding for the cDNA of various trypsin-like serine proteases or an empty vector. Transfected cells were harvested at 48 h post transfection (p.t.) and subsequently subjected to SDS-PAGE and Western Blot analysis as described below. For the immunofluorescence staining of S-mediated cell–cell fusion, HeLa cells were grown to near confluency on glass cover slips coated with type I collagen from rat tail (Corning, Corning, NY, USA, 354236). The cells were then co-transfected with 0.8 µg of expression plasmids coding for the cDNA sequence of SARS or MERS S, respectively, 0.8 µg of pCDNA3.1(+)-ACE2-HA or pCMV3-DPP4-cHA and 15 ng of expression plasmids coding for the cDNA of various trypsin-like serine proteases or an empty vector, as described above.

### 2.9. SDS-PAGE and Western Blot Analysis

Infected Calu-3 cells or transfected HeLa cells were harvested by low-speed centrifugation, washed with PBS, and lysed with CellLytic M buffer (Sigma-Aldrich, St. Louis, MO, USA, C2978) supplemented with a protease inhibitor cocktail (Sigma-Aldrich, St. Louis, MO, USA, P8340). The samples were resuspended in reducing SDS-PAGE sample buffer and heated at 95 °C for 10 min. The samples derived from infected cells or supernatant were heated to 100 °C for 10 min twice, before being subjected to an 8% (S) or 12% (HA) polyacrylamide gel for SDS-PAGE. Subsequently, the proteins were transferred to a polyvinylidene difluoride (PVDF) membrane (Cytiva, Marlborough, MA, USA, 10600023) and detected using the primary and species-specific HRP-conjugated secondary antibodies described above. A ChemiDoc XRS system with Image Lab software (version 6.1, Bio-Rad, Hercules, CA, USA) was used for visualization.

### 2.10. Immunofluorescence Staining and Microscopy

HeLa cells transiently co-expressing C-terminally FLAG-tagged SARS- or MERS-CoV S and their respective receptors ACE2 or DPP4, and various trypsin-like proteases were thoroughly washed with PBS and fixed with a 1:1 mixture of methanol and acetone at 24 h p.t. To visualize S-mediated cell–cell fusion, the cells were stained with a FLAG-specific primary antibody and the species-specific fluorophore-conjugated secondary antibody described above. DAPI (ThermoFisher, Waltham, MA, USA, D1306) was used for the counter staining of nuclei. The glass cover slips were mounted on glass slides with Fluoroshield (Sigma-Aldrich, St. Louis, MO, USA, F6937). Microscopic analysis was performed using the Leica OMI6000 B fluorescence microscope with LAS X software (version 3.7.0.20979, Leica, Wetzlar, Germany).

### 2.11. Cell Viability Assay

Calu-3 cells grown to near confluency in 96-well plates were treated with PPMO for 24 h, as described above, and were then further incubated with or without addition of protease inhibitors for another 24 h. Infection medium containing 10% ethanol was used as a positive control. The cell viability was then analysed using the CellTiter-Glo^®^ 2.0 Cell Viability Assay Kit (Promega, Madison, WI, USA, G9241) according to the manufacturer’s protocol.

### 2.12. Synthesis of Fluorescence Resonance Energy Transfer (FRET) Substrates

The peptides were synthesized by automated solid-phase peptide synthesis on a Syro 2000 synthesizer (MultiSynTech GmbH, Witten, Germany) using ~120 mg of Rink Amide MBHA resin (loading 0.73 mmol/g) for each 2 mL reaction vessel and a standard Fmoc-protocol with double couplings (approximately fourfold excess of Fmoc amino acid, HOBt and HBTU, respectively, and 8 equiv. DIPEA, 2 × 2 h coupling time), as described recently [54]. After the final coupling of Boc-2-aminobenzoic acid, the resin was washed with 20% piperidine in DMF (5 and 15 min) to remove any potential acylation on the 3-nitrotyrosine [55]. The peptides were cleaved from the resin and deprotected by a mixture of TFA/triisopropylsilane/water (95/2.5/2.5, *v*/*v*/*v*) over 2 h at RT, followed by precipitation in cold diethyl ether. All peptides were purified by preparative reverse-phase HPLC to more than 95% purity based on the detection at 220 nm and finally obtained as lyophilized TFA salts.

All analytical HPLC experiments were performed on a Primaide system (VWR, Hitachi; column: NUCLEODUR C18 ec, 5 μm, 100 Å, 4.6 mm × 250 mm, Macherey-Nagel, Allentown, PA, USA), using 0.1% (*v*/*v*) TFA in water (solvent A) and 0.1% (*v*/*v*) TFA in acetonitrile (solvent B) as eluents. A linear gradient with an increase of 1% solvent B/min at a flowrate of 1 mL/min was applied. Purification by preparative HPLC was performed on a Knauer system (Knauer Azura P 2.1L with HyperShear static mixer; detector: Knauer UVD 2.1L; collector: Foxy R1, column: NUCLEODUR C18 ec, 5 μm, 100 Å, 32 mm × 250 mm, Macherey-Nagel, Allentown, PA, USA) with the same solvents as those used for the analytical experiments. In this case, a linear gradient with an increase of 0.5% solvent B/min was applied. Detection for both analytical and preparative experiments was performed at 220 nm. Mass spectrometry was performed on a QTrap 2000 ESI spectrometer (Applied Biosystems, Norwalk, CT, USA).

### 2.13. Enzyme Kinetic Measurements with Recombinant TMPRSS2

Recombinant soluble human TMPRSS2 (aa 109-492; mutation SRQSR255→ DDDDK255) was kindly provided by Amy DeRocher (Seattle Structural Genomics Center for Infectious Disease, Seattle, WA, USA). TMPRSS2 was prepared in SF9 insect cells and activated by enteropeptidase according to a protocol described in [56]. The protein concentration was determined by measuring the absorbance at 280 nm using a calculated extinction coefficient of 2.292. The measurements were performed in black 96-well plates (Nunc) at RT using a microplate reader (Spark, Tecan, Houston, TX, USA) at λ_ex_ 320 nm and λ_em_ 420 nm. Each well contained 20 μL of the substrate solution (190 µM, dissolved in water) and 150 μL of buffer (50 mM Tris at pH 8.0 containing 154 mM NaCl and 1 g/L PEG8000). The measurements were started by the addition of 20 μL of TMPRSS2 solution (0.114 nM in assay) [57]. Each 15 s, 30 data points were collected, and the steady-state rates were calculated from the slopes of the progress curves.

## 3. Data Analysis

Data are shown as mean values + SD or SEM for at least three biologically independent experiments, as indicated. Statistical significance was calculated using GraphPad Prism (version 10). To perform the statistical analysis of the viral growth curves, the raw data were transformed to the percentage of untreated or DMSO-treated controls for each time point. Microscopic images were further analysed using ImageJ 1.54f software (version 2.14.0).

## 4. Results

### 4.1. SARS-CoV S and MERS-CoV S Differ in Their Cleavability by TMPRSS2

**Figure 2:**
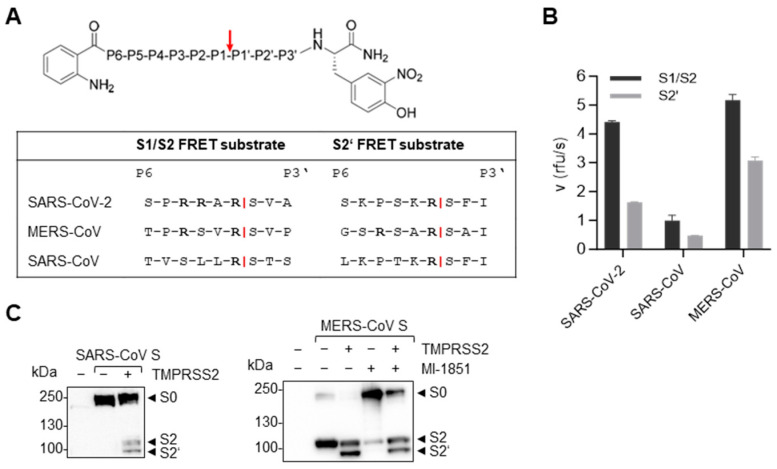
**Efficiency of TMPRSS2 cleavage of SARS-CoV and MERS-CoV S.** (**A**) P6-P3′ amino acid sequences of the synthesized and analysed FRET substrates representing the CoV S cleavage site motifs, which all contain an N-terminal o-aminobenzoyl fluorophore and a C-terminal Tyr(3-NO_2_)-NH_2_ as a quenching residue. The TMPRSS2 cleavage site is indicated by a red arrow. (**B**) The cleavage efficiency of the FRET substrates by recombinant TMPRSS2 was measured in an enzyme kinetic assay. The data shown are the mean values + SD based on three independent measurements with three independent weights of the substrates. (**C**) HeLa cells were transfected with plasmids encoding C-terminally FLAG-tagged SARS-CoV or MERS-CoV S, and TMPRSS2. Simultaneously, HeLa cells were treated with 50 µM MI-1851 or remained untreated. At 48 h after transfection and inhibitor treatment, the cell lysates were subjected to SDS-PAGE and Western blot analysis with an antibody targeting the S2 subunit of SARS-CoV S or the C-terminal FLAG-tag of MERS-CoV S. The data shown are the representative results of three independent experiments.

Given the established involvement of TMPRSS2 in the cleavage and activation of all three zoonotic CoVs, we first aimed to compare the cleavage efficiency of the S cleavage sites of the different viruses by TMPRSS2. The S cleavage site motifs of SARS-CoV, MERS-CoV and SARS-CoV-2 differ considerably. While SARS-CoV-2 S harbours a multibasic sequence R-R-A-R↓ at the S1/S2 site, the S1/S2 and S2′ sites of MERS-CoV S comprise the dibasic motifs R-S-V-R↓ and R-S-A-R↓, respectively (Figure 2A). Both cleavage sites of SARS-CoV S, as well as the S2′ site of SARS-CoV-2, contain a single arginine residue at the cleavage site R↓, but they all differ in the preceding amino acid sequence, which could potentially influence cleavability by TMPRSS2. To test the efficiency of cleavage by TMPRSS2, we designed and synthesized FRET substrates representing the S1/S2 and the S2′ site motifs, including six residues upstream (P6) and three residues downstream (P3′) of the cleavage sites of SARS-CoV-2, MERS-CoV and SARS-CoV (Figure 2A). Enzyme kinetic measurements with recombinant TMPRSS2 confirmed that both cleavage sites of SARS-CoV-2 S could be efficiently processed by TMPRSS2, although furin has been shown to be responsible for the cleavage of the S1/S2 site during infection [31,32]. The dibasic cleavage sites of MERS-CoV S were both processed by TMPRSS2 with high efficiency, especially the S1/S2 motif, while SARS-CoV S was processed with relatively low efficiency (Figure 2B).

We thus went on to confirm the differences in the relative sensitivity of MERS-CoV and SARS-CoV S to cleavage by TMPRSS2, by using transient co-expression of S with a C-terminal FLAG-tag and the protease in HeLa cells. Western blot analysis of the cell lysates at 48 h p.t. with an antibody targeting the FLAG-tag revealed that, consistent with the FRET assay results, only a fraction of SARS-CoV S was processed into its cleavage products S2 and S2′ in the presence of TMPRSS2 (Figure 2C, left panel). MERS-CoV S, on the other hand, was efficiently processed at the S2′ site by co-expressed TMPRSS2, while the S1/S2 cleavage was carried out by a different endogenous protease in HeLa cells (Figure 2C, right panel). When HeLa cells were treated with the furin inhibitor MI-1851 during transfection, the majority of S was detected as non-cleaved precursor S0. Therefore, we identified furin as the protease responsible for the S1/S2 cleavage of MERS-CoV S. The transient expression of TMPRSS2 in the presence of the furin inhibitor, however, revealed that TMPRSS2 was able to process both cleavage sites of MERS-CoV S and could compensate for the lack of furin. This finding was consistent with the high efficiency observed for MERS-CoV S cleavage by TMPRSS2 in the FRET assay (Figure 2B).

### 4.2. SARS-CoV S but Not MERS-CoV S Can Be Functionally Activated by mTMPRSS4 and mTMPRSS13

Previous studies have addressed the ability of trypsin-like serine proteases other than TMPRSS2 to activate the S of various CoVs. TMPRSS13 was found to cleave both MERS- and SARS-CoV S at the S2′ site, activating S for membrane fusion and enhancing the cellular uptake and replication of SARS-CoV [58,59,60]. HAT was also reported to cleave SARS- and MERS-CoV S at the S1/S2 site [43,58,61]. HAT facilitated MERS-CoV S-mediated virus–cell fusion [58], but there are contradictory findings concerning its ability to facilitate SARS-CoV S-mediated cathepsin-independent virus–cell fusion and viral replication [58,60,61]. Interestingly, TMPRSS4 promoted S-mediated cell–cell fusion in a phenotypically similar manner to that of TMPRSS2, even though no S cleavage products were detectable via Western blot [62]. As we observed striking differences between the TMPRSS2 cleavage efficiency of SARS- and MERS-CoV S (Figure 2), we next sought to compare the cleavability of SARS- and MERS-CoV S by the TMPRSS2-related trypsin-like serine proteases HAT, TMPRSS4 and TMPRSS13. Additionally, we analysed S-mediated cell–cell fusion. Since the plasmids for human TMPRSS13 and TMPRSS4 were not available, we used plasmids encoding murine TMPRSS4 and TMPRSS13 [49]. Murine and human TMPRSS4 and TMPRSS13 share 77% and 83% amino acid identity, respectively, and the catalytic triads are conserved between the orthologues.

**Figure 3:**
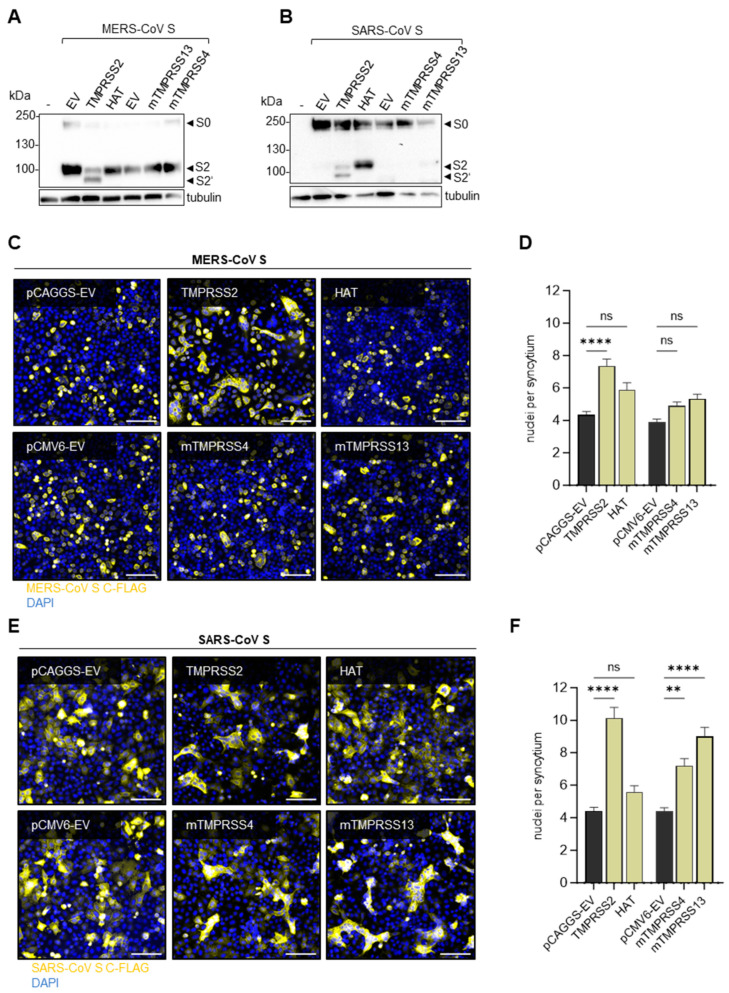
**Activation of SARS-CoV and MERS-CoV S by TMPRSS2 and further host proteases.** (**A**,**B**) HeLa cells were co-transfected with plasmids coding for MERS-CoV S (**A**) or SARS-CoV (**B**) with a C-terminal FLAG-tag and plasmids coding for human TMPRSS2 or HAT or murine TMPRSS4 or TMPRSS13. Cell lysates were analysed via SDS-PAGE at 48 h p.t. Western blot analysis was performed using an antibody targeting the C-terminal FLAG-tag. Tubulin was used as a loading control. The data shown are representative of three individual experiments. EV = empty vector. (**C**,**E**) HeLa cells co-expressing MERS-CoV (**C**) or SARS-CoV (**E**) S, as well as the respective receptors DPP4 or ACE2, were incubated for 48 h. After fixation, the cells were stained with a primary antibody targeting the C-terminal FLAG-tag of S and a fluorescence-coupled secondary antibody. DAPI was used to stain the nuclei. Representative images of three independent experiments are shown. The scale bar represents 100 µM. (**D**,**F**) Quantification of MERS-CoV S (**D**) or SARS-CoV (**F**) mediated cell–cell fusion. For each condition, ten randomly taken images were analysed by counting the nuclei per syncytium (with at least three nuclei). The data shown are the mean values + SEM of three independent experiments. Statistical significance was determined with a one-way ANOVA followed by Šídák’s multiple comparisons test. ns = not significant, ** = *p* < 0.01, **** = *p* < 0.0001.

MERS-CoV S or SARS-CoV S, respectively, were co-expressed with HAT, mTMPRSS4 or mTMPRSS13 in HeLa cells. Western blot analysis at 48 h p.t. with FLAG-tag-specific antibodies revealed that MERS-CoV S was processed at the S2′ cleavage site only in the presence of TMPRSS2; meanwhile, HAT, mTMPRSS4 and mTMPRSS13 did not have an effect on S cleavage (Figure 3A). Likewise, the SARS-CoV S cleavage products S2 and S2′ were only detected upon co-expression of TMPRSS2 (Figure 3B). Interestingly, the co-expression of HAT or, to a lesser extent, mTMPRSS13, resulted in a cleavage product roughly the size of S2, but no S2′, while no cleavage products at all were detected with co-expressed mTMPRSS4. To investigate whether there was still a minimal amount of cleavage sufficient for S activation, we performed cell–cell fusion assays with HeLa cells transiently expressing MERS-CoV or SARS-CoV S, as well as their respective cellular receptors, DPP4 or ACE2, and the different proteases. At 48 h p.t., syncytia formation was analysed via immunofluorescence staining of S by targeting the FLAG-tag. Only S processed at both cleavage sites was able to mediate membrane fusion with neighbouring cells expressing the appropriate receptor. As expected from the previous results, MERS-CoV S was able to mediate cell–cell fusion only in the presence of TMPRSS2 (Figure 3C). In contrast to that, the S of SARS-CoV mediated fusion when co-expressed with TMPRSS2, mTMPRSS4 and mTMPRSS13 (Figure 3E). Individually, only HAT was unable to activate the SARS-CoV S fusion capacity. The quantification of syncytia formation by counting the nuclei per syncytium supported the finding that only TMPRSS2 significantly enhanced cell–cell fusion mediated by MERS-CoV S, although a slight increase in syncytia size was evident in the presence of HAT, mTMPRSS4 and mTMPRSS13 (Figure 3D). SARS-CoV S-mediated fusion was significantly enhanced by TMPRSS2, mTMPRSS4 and mTMPRSS13 (Figure 3F). These findings suggest that MERS-CoV S activation is primarily dependent on TMPRSS2 whereas SARS-CoV S can achieve activation by various other proteases as well.

### 4.3. Multicycle Replication of MERS-CoV Is More Dependent on TMPRSS2 than SARS-CoV

When S is produced exogenously in vitro, it is transported to the plasma membrane via the classical secretory pathway. During its natural virus life-cycle, however, CoVs bud into the endoplasmic reticulum–Golgi intermediate compartment (ERGIC) [63]. The cleavage events of CoV S may thus differ in timing and location during plasmid-generated transient expression compared to authentic infection. Hence, it is crucial to investigate the impact of activating proteases not only during transient co-expression but also during authentic viral infection. To mimic the in vivo target cells of CoVs, we used Calu-3 human airway cells that endogenously express TMPRSS2. Indeed, TMPRSS2 has been shown to be the primary activating protease of IAV HA with a monobasic cleavage site in these cells [23,24]. We used the PPMO T-ex5 to knockdown the expression of catalytically active TMPRSS2 as previously described [52]. Briefly, T-ex5 masks the spliceosome acceptor site of exon 5 of the pre-mRNA of TMPRSS2, resulting in the production of a truncated protein lacking proteolytic activity. The inhibition of TMPRSS2 by T-ex5 is therefore highly selective, in contrast to conventional broad-spectrum serine-protease inhibitors such as camostat mesylate, which target a variety of TTSPs.

**Figure 4:**
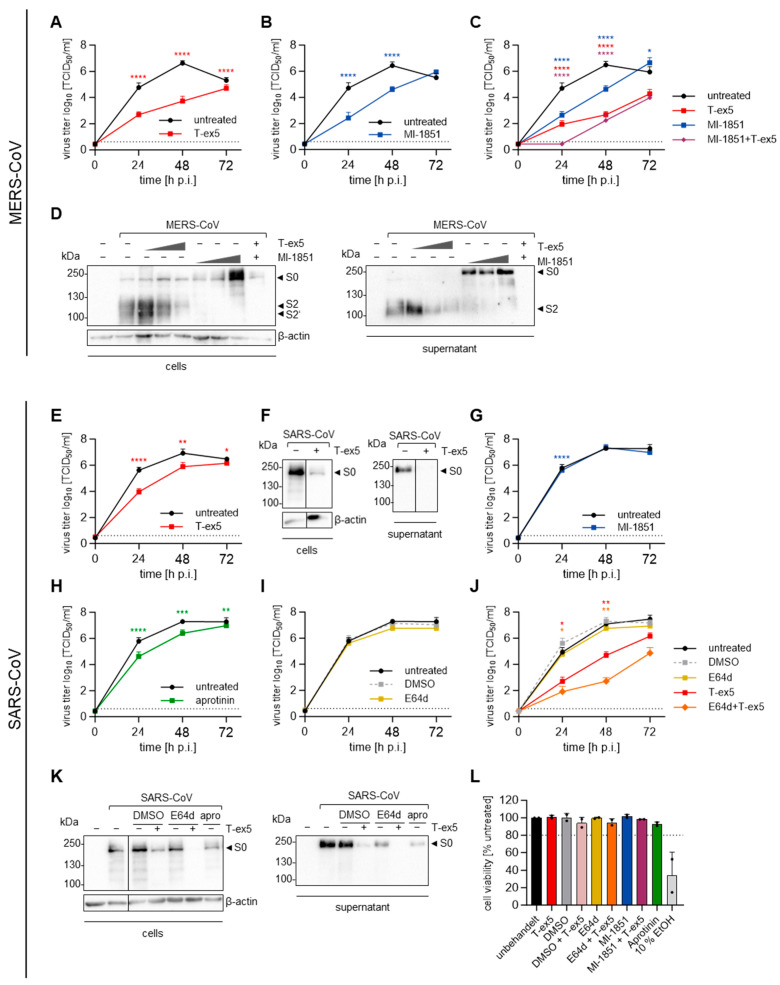
**Protease dependency of the multicycle replication of SARS-CoV and MERS-CoV in Calu-3 cells.** (**A**–**C**) Calu-3 cells were treated with or without 25 µM T-ex5 for 24 h. The cells were then infected with MERS-CoV at an MOI of 0.001. After inoculation with the virus for 1 h, cells were treated with 50 µM MI-1851 or remained untreated. Viral titers in the supernatant sampled at the indicated time points were determined via TCID_50_ endpoint titration. The data shown are the mean + SD of three independent experiments. The dotted lines indicate the limit of detection (LOD). (**D**) Calu-3 cells treated with 5, 15 or 25 µM T-ex5 or left untreated were infected with MERS-CoV at an MOI of 1 and treated with 5, 10 or 50 µM MI-1851 or no inhibitor after 1 h inoculation. At 24 h p.i., the cell lysates and supernatant were subjected to SDS-PAGE, and Western blot analysis was performed with antibodies targeting the S2 subunit of MERS-CoV S. For the cell lysates, ß-actin was stained as a loading control. The data shown are the representative results of three independent experiments. (**E**,**G**–**J**) Calu-3 cells pre-treated with 25 µM T-ex5 for 24 h or left untreated were infected with SARS-CoV at an MOI of 0.001. After 1 h, the cells were washed and treated with 50 µM MI-1851 (**G**), 50 µM aprotinin (**H**), 50 µM E64d or DMSO (**I**,**J**). The supernatant was sampled at 0, 24, 48 and 72 h p.i. and the viral titers were determined via TCID_50_ endpoint titration. The experiments were performed three times and the data shown are mean + SD. The dotted lines indicate the LOD. (**F**,**K**) Calu-3 cells infected with SARS-CoV at an MOI of 1 were either pre-treated with 25 µM of T-ex5 or treated with 50 µM of the different inhibitors at 1 h after inoculation. After an additional 24 h, the cell lysates and supernatants were harvested for SDS-PAGE. Western blot analysis was performed with an antibody targeting the S2 subunit of SARS-CoV S and ß-actin was used as a loading control. The results are representative of three individual experiments. (**L**) Calu-3 cells were treated with 25 µM T-ex5 and/or 50 µM of the different inhibitors, or remained untreated, as in the previous experiments. At 72 h after inhibitor treatment, the cell viability was assessed and untreated cells were set to 100%, and inhibitors or combinations with over 80% cell viability (dotted line) were considered non-toxic. Treatment with 10% ethanol for 24 h served as a positive control. The data shown are the mean + SD of two independent experiments each performed in a technical triplicate. To determine the statistical significance of the differences in viral titers compared to untreated or DMSO-treated controls, an unpaired *t*-test (**A**,**B**,**E**,**G**–**I**) or a one-way ANOVA followed by Tukey’s multiple comparisons test (**C**,**J**) was performed for each individual time point. ns = not significant, * = *p* < 0.05, ** = *p* < 0.01, *** = *p* < 0.001, **** = *p* < 0.0001.

To study the multicycle replication of SARS- and MERS-CoV in Calu-3 cells, cells were either pre-treated with T-ex5 for 24 h or were left untreated. The cells were then infected with a low MOI (0.001) for 72 h. At indicated time points, the viral titers were determined in the supernatant by TCID_50_ endpoint titration. We observed that MERS-CoV viral titers were significantly reduced up to 1000-fold in T-ex5-treated cells (Figure 4A). Since we found that S1/S2 cleavage in HeLa cells was carried out by furin (Figure 2C), we next analysed MERS-CoV replication in the presence of the furin inhibitor MI-1851. Calu-3 cells were infected as above for 1 h, and, after the removal of the inoculum, the inhibitor was added in replenished medium. We noted a significant decrease in viral titers of about 100-fold (Figure 4B). Since the inhibitors were not administered repeatedly and the virus replication in the untreated cells had reached its maximum, the virus titers in treated and untreated cells equalised after 72 h. Inhibiting the action of both TMPRSS2 and furin simultaneously, via T-ex5 and MI-1851, showed an additive antiviral effect, further reducing the MERS-CoV viral titer at 24 h p.i. to below the limit of detection (Figure 4C). These data indicate that both proteases are critical for MERS-CoV replication in Calu-3 cells. To confirm that the reduced viral titers were due to interference with S cleavage, we treated Calu-3 cells with different concentrations of T-ex5 or MI-1851 and infected them with an MOI of 1. Western blot analysis of the infected cells at 24 h p.i., using an antibody targeting the MERS-CoV S S2 subunit, revealed that T-ex5 treatment led to reduced levels of the S2′ cleavage product in a dose-dependent manner, while S cleavage products were undetectable in the presence of MI-1851 (Figure 4D, left panel). Hence, surprisingly, the ability of TMPRSS2 to process the S1/S2 cleavage site and compensate for furin, as observed during transient co-expression (Figure 2B,C, right panel), was not noted during multicycle replication in Calu-3 human airway cells. In the supernatant of untreated infected cells, only fully cleaved S was detected, while T-ex5 treatment reduced the cleavage products in a dose-dependent manner. However, mostly non-cleaved precursor S0 was present when the cells were treated with MI-1851, again highlighting that TMPRSS2 cannot compensate for furin during authentic infection in human airway cells (Figure 4D, right panel).

SARS-CoV replication was reduced significantly, but much weaker compared to the inhibition of MERS-CoV replication when the cells were treated with T-ex5 for 24 h before infection (Figure 4E), consistent with the comparatively low efficiency of SARS-CoV S cleavage by TMPRSS2 observed in the FRET and transient co-expression assays (Figure 2B,C). We also performed Western blot analysis of cells and supernatants after infection with an MOI of 1 for 24 h in the absence of active TMPRSS2, using an antibody targeting the S2 subunit of SARS-CoV S. The immunoblots revealed that the 100-fold reduction in viral titers was accompanied by a strong reduction in the detection of total S in infected cells and no detection of S in the supernatant (Figure 4F). The furin inhibitor MI-1851 had a significant although marginal effect on SARS-CoV replication at 24 h p.i. but did not affect the final virus titers, as expected (Figure 4G). As we had previously observed that SARS-CoV S can employ mTMPRSS4, mTMPRSS13 and potentially other serine proteases for its activation, we next tested the overall involvement of serine proteases in SARS-CoV multicycle replication in Calu-3 cells, via treatment with the natural broad-spectrum serine protease inhibitor aprotinin from bovine lungs. Yet, aprotinin showed no more inhibitory effect on SARS-CoV replication than T-ex5, with a reduction in titers of only slightly more than 10-fold (Figure 4H). It has been shown that SARS-CoV-2 S is processed by endosomal cathepsins in the absence of TMPRSS2 [29,32], and cathepsin L was found to cleave SARS-CoV S near the S1/S2 site [34]. Hence, we analysed the multicycle replication of SARS-CoV in Calu-3 cells treated with the endosomal cathepsin inhibitor E64d and observed no difference in viral titers between treated and untreated cells (Figure 4I). When the cells were treated with T-ex5 and E64d simultaneously, however, we observed a strong 10,000-fold reduction in virus titers compared to the controls at 48 h p.i., representing an additive effect of 100-fold (Figure 4J). This confirmed that SARS-CoV S cleavage carried out by endosomal cathepsins is an alternative to cleavage by TMPRSS2, as has previously been demonstrated by Kawase et al. [64]. Correspondingly, we observed that the amount of S detectable in cells infected at an MOI of 1 for 24 h was further reduced when both T-ex5 and E64d were present together compared to treatment with either agent (Figure 4K, left panel). The amount of S in the virus-containing supernatant was markedly reduced with E64d treatment alone and no S was detected when both TMPRSS2 and endosomal cathepsin inhibitors were used (Figure 4K, right panel). To exclude the possibility that the reduction in viral titers was due to the cytotoxic effects of the various inhibitors, we treated Calu-3 cells under the same conditions used in the infection experiments and analysed cell viability at the time point equaling 72 h p.i., with 10% ethanol serving as a positive control reagent for cytotoxicity. As expected, we found that none of the protease-directed inhibitors had an impact on cell viability under any of the treatment conditions (Figure 4L).

### 4.4. TMPRSS2 Is the Main Activating Protease of H1N1/1918 and H13 While Activation of H12 and H17 Is TMPRSS2-Independent

**Figure 5:**
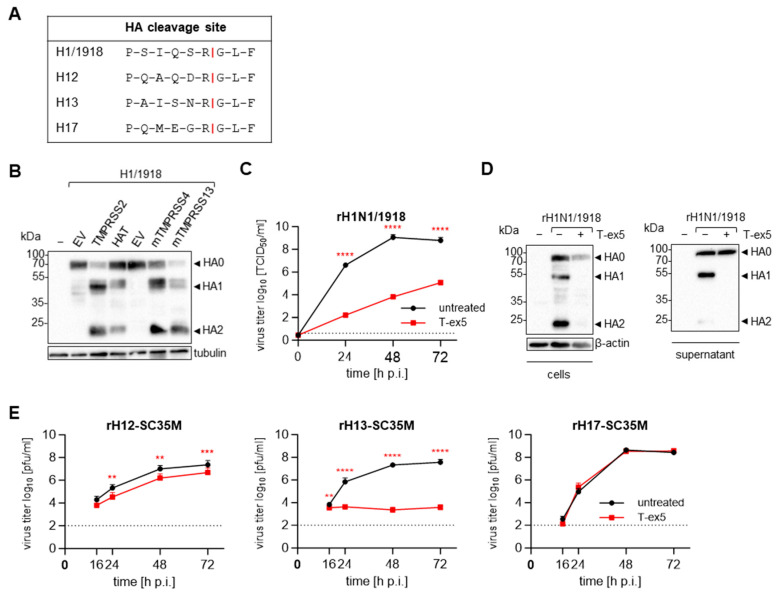
**TMPRSS2 dependency of H1N1/1918, H12, H13 and H17.** (**A**) Sequence alignment of the cleavage sites from different HAs. (**B**) HEK293 cells transiently expressing the H1/1918 and human TMPRSS2 or HAT, or murine TMPRSS4 or TMPRSS13 were subjected to SDS-PAGE at 48 h p.t. Western blot analysis was performed with an antibody targeting H1. Tubulin was stained as a loading control. The result shown is representative of three independent experiments. EV = empty vector. (**C**) Calu-3 cells were treated with 25 µM T-ex5 for 24 h before being infected with rH1N1/1918 at an MOI of 0.001. Viral titers in the supernatant sampled at the indicated time points were determined via TCID_50_ endpoint titration. The data shown are the mean + SD of three individual experiments. The dotted lines indicate the LOD. (**D**) The cell lysates and supernatants (SN) of Calu-3 cells treated with 25 µM of T-ex5 for 24 h and then infected with rH1N1/1918 at an MOI of 1 for another 24 h were subjected to SDS-PAGE, and an antibody targeting H1 was used for the Western blot analysis. ß-actin was used as a control. The result is representative of three independent experiments. (**E**) Calu-3 cells pre-treated with 25 µM were infected with recombinant viruses containing avian H12 or H13 or bat-derived H17 and sharing the other seven gene segments of SC35M at a low MOI of 0.01–0.0001. The supernatant of infected cells was harvested at indicated time points and the viral titers were determined via an immune plaque assay. The experiments were performed in triplicate and the data shown are mean + SD. The dotted lines indicate the LOD. (**C**,**E**) Statistically significant differences in viral titers at each time point compared to untreated controls were determined by performing unpaired t-tests. ns = not significant, ** = *p* < 0.01, *** = *p* < 0.001, **** = *p* < 0.0001.

The HA of the 1918 H1N1 virus has been demonstrated to be a factor in the enhanced virulence of this highly pathogenic virus [65]. The amino acid sequence of the HA cleavage site is shown in Figure 5A. While the cleavage of HAs with a monobasic cleavage site, including the H1 from the 2009 IAV pandemic, can be carried out by several trypsin-like serine proteases in various in vitro settings [66], the 1918 HA has so far only been shown to be processed by TMPRSS2 and TMPRSS4 [67,68]. We therefore studied whether the known HA-activating proteases HAT and mTMPRSS13 are able to cleave the 1918 HA upon co-expression in HEK293 cells as well. The cells were transfected with plasmids encoding the 1918 HA and each of the four proteases mentioned above, respectively. Cell lysates were collected at 48 h p.t. for SDS-PAGE and Western blot detection of H1 HA. Interestingly, all four proteases were able to efficiently process the H1N1/1918 HA into its subunits (Figure 5B). Although many proteases have been documented to proteolytically process HA with monobasic cleavage sites upon co-expression in vitro, we previously showed that TMPRSS2 is the major activating protease for most IAV subtypes during infection of Calu-3 human airway cells [23,24,52]. Thus, we next investigated the multicycle replication of the recombinant H1N1/1918 in Calu-3 cells in the absence or presence of TMPRSS2. As described above, Calu-3 cells were pre-treated with T-ex5 for 24 h to knockdown the expression of catalytically active TMPRSS2. Subsequently, the cells were infected with a low MOI (0.001) and the viral titers in the supernatant were determined by TCID_50_ endpoint titration. Strikingly, viral titers were reduced 100,000-fold in T-ex5-treated cells compared to untreated controls at 48 h p.i. (Figure 5C). Western blot analysis of Calu-3 cells infected with H1N1/1918 at an MOI of 1 for 24 h with an HA-specific antibody revealed that the dramatic decrease in viral titers is associated with a complete loss of the HA cleavage products HA1 and HA2 (Figure 5D, left panel). In agreement with this result, low amounts of viral protein and only non-cleaved precursor HA0 could be detected in the supernatant containing virions released from infected cells (Figure 5D, right panel), emphasizing the essential role of TMPRSS2 in HA activation and viral replication of H1N1/1918.

In addition to the 1918 HA, the avian HA subtypes H12 and H13, as well as the bat-derived H17, have not yet been evaluated for dependency on TMPRSS2 in human airway cells. All three HA subtypes possess a monobasic cleavage site with various preceding amino acid sequences (Figure 5A). To study the multicycle replication mediated by these HAs, we generated recombinant 1:7 reassortant viruses harbouring either H12, H13 or H17 while sharing the other seven gene segments of an avian- and mouse-adapted strain of highly pathogenic H7N7 (SC35M) [69]. We again treated Calu-3 cells with or without T-ex5 for 24 h before infecting the cells with an MOI of 0.01 to 0.0001, and analysed the viral titers at indicated time points via an immune plaque assay. While rH13-SC35M was found to require TMPRSS2 for replication, similar to most IAV subtypes, the replication of rH12-SC35M was only slightly reduced by T-ex5 treatment and the bat-derived rH17-SC35M exhibited TMPRSS2-independent replication in Calu-3 cells, in a manner observed to date only with IBV and to a lesser extent with H16 (Figure 5E) [23,24]. These findings emphasize the need to investigate the effect of further HA-activating proteases that may play a role in the multicycle replication of TMPRSS2-independent viruses.

In summary, we demonstrated that the highly pathogenic respiratory viruses SARS-CoV, MERS-CoV and IAV H1N1/1918 differ in their TMPRSS2 dependency (Table 1). H1N1/1918 HA cleavage and multicycle replication were dramatically decreased in the absence of TMPRSS2 (Figure 5C,D). MERS-CoV proved to be similarly TMPRSS2-dependent, yet furin was found to be additionally required for efficient viral replication and S cleavage (Figure 4A–D). Despite the presence of similar amino acid sequences at the MERS-CoV S S1/S2 and S2′ sites, the two proteases could not compensate for each other during MERS-CoV infection of Calu-3 cells (Figure 4C). Interestingly, SARS-CoV S, albeit harbouring a monobasic S1/S2 cleavage site and a single arginine at the S2′ site, was found to depend far less on the presence of catalytically active TMPRSS2 than MERS-CoV and SARS-CoV-2 (Figure 4E). Instead, SARS-CoV S could employ several other trypsin-like serine proteases for functional activation (Figure 3B,E,F), and used cathepsin-mediated late entry during infection when TMPRSS2 was absent (Figure 4E). In addition, we observed a TMPRSS2-independent phenotype for H12 and H17-mediated multicycle replication in Calu-3 cells that has, to date, been reported only for influenza B virus (Figure 5E) [23].

## 5. Discussion

In this study, we found that IAVs and CoVs differ in their dependency on TMPRSS2 for structural protein cleavage and the virus activation it engenders. We observed that TMPRSS2 was able to process S of SARS-CoV and MERS-CoV at both cleavage sites (Figure 2B,C). However, there were clear differences in the cleavage efficiency of the FRET assay and upon co-expression. Further, during infection of Calu-3 human airway cells, we found that MERS-CoV replication was highly dependent on TMPRSS2, whereas, to our surprise, SARS-CoV viral titers were only moderately reduced when catalytically active TMPRSS2 was not present in sufficient quantities (Figure 4A,E). To date, it has generally been assumed that SARS-CoV, bearing S with a monobasic S1/S2 cleavage site and a single arginine at the S2′ site, would be fully activated by TMPRSS2. Our results were therefore unexpected. It has been reported that TMPRSS2 knockout mice were protected from severe pathogenesis upon infection with MERS-CoV or SARS-CoV [44]. Interestingly, Iwata-Yoshikawa et al. observed that the impact on pathogenesis was considerably greater for MERS-CoV infection than it was for SARS-CoV in the TMPRSS2 knockout mice, indicating a more pronounced dependency on TMPRSS2 for MERS-CoV, consistent with our findings.

We found that cathepsins were employed by SARS-CoV in the absence of TMPRSS2 in human airway cells (Figure 4J). For MERS-CoV, on the other hand, previous studies suggested a cell-type-dependent difference in the involvement of endosomal cathepsins, with the pathway being restricted in Calu-3 human airway cells [37,42,43]. Correspondingly, we could show that MERS-CoV was not able to use endosomal cathepsins for S cleavage when both TMPRSS2 and furin were unavailable in these cells, as viral titers were reduced to below the limit of detection at 24 h p.i. (Figure 4C). These data indicate a clear difference between the ability of the two viruses to employ cathepsins for S activation. It has been shown that TMPRSS2-mediated entry at the plasma membrane, rather than endosomal entry, is preferred by SARS-CoV-2 [70]. Using the early entry route may enable the virus to evade the interferon-induced-transmembrane (IFITM)-mediated blocking of viral membrane fusion in the endosome [71,72]. Therefore, employing TMPRSS2 instead of cathepsins as the primary activating protease may be beneficial for virus infectivity in humans, suggesting that SARS-CoV may have been less adapted to humans than SARS-CoV-2. Clinical isolates of HCoV-229E and HCoV-OC43 have also been shown to prefer activation by TMPRSS2 over cathepsins [73,74]. At this point, it should be mentioned that there is a controversial discussion about whether cathepsins play a role in human CoV infections or primarily contribute to virus activation in cell cultures in vitro.

In addition to the interesting differences in the usage of TMPRSS2 and cathepsins, we observed differences in SARS- and MERS-CoV S cleavage by mTMPRSS4 and mTMPRSS13. We found that both proteases were able to functionally activate SARS-CoV S but not MERS-CoV S for cell–cell fusion (Figure 3C–F). We observed that HAT and mTMPRSS13 were able to process SARS-CoV S, resulting in a cleavage product with a size similar to S1/2. Interestingly, this was not sufficient to enable S-mediated cell–cell fusion in case of HAT (Figure 3B,E,F), contradicting previous findings [61], but supported cell–cell fusion in mTMPRSS13-expressing cells. Calu-3 cells express very little TMPRSS13, but high levels of TMPRSS4 [23]. Yet, SARS-CoV replication was no more reduced after treatment of the cells with the broad-range serine-protease inhibitor aprotinin than it was with the TMPRSS2-specific PPMO T-ex5 (Figure 4E,H), suggesting that TMPRSS4 and other endogenous serine proteases are not involved in SARS-CoV S activation during infection in these cells. This again indicates that cathepsins play an important role in SARS-CoV activation in Calu-3 cells. However, primary human bronchial epithelial cells have been shown to express a higher number of serine proteases and at higher levels than are present in Calu-3 cells, so TMPRSS13 and other proteases may contribute to SARS-CoV S activation in the human host [23].

The role of furin in MERS-CoV S cleavage and viral replication has been a matter of controversy. On the one hand, there is evidence that MERS-CoV S is cleaved by furin and that the protease is essential for infection in different cell types [37,38,75]. On the other hand, several studies have shown that furin was dispensable for S-mediated cell–cell fusion, entry and replication [38,39]. While the furin consensus cleavage site is considered to be R-X-R/K-R↓, several substrates with non-basic amino acids in the P2 position have been identified, e.g., Shiga toxin (R-V-A-R↓) or *Pseudomonas aeruginosa* exotoxin A (R-Q-P-R↓) [76]. A closer look at the dibasic cleavage sites of MERS-CoV S and their cleavability by furin lead to contradictory results. Örd et al. found the R-X-X-R↓ motif not sufficient for in vitro furin cleavage [41], whereas Jaimes et al. demonstrated efficient cleavage of the S2′ site motif by furin [40]. Relatedly, we showed in a previous study that the S1/S2 site of MERS-CoV is only poorly processed by furin in comparison to the S1/S2 site of SARS-CoV-2 [31]. Nevertheless, the results of the study here show that furin is not only able to cleave the S1/S2 site of MERS-CoV S during transient expression of S in HeLa cells, but that it also plays a critical role during the infection of Calu-3 human airway cells, as viral titers were reduced more than 100-fold in the presence of a furin inhibitor (Figure 2C and Figure 4B). Blocking furin in addition to TMPRSS2 led to a reduction in viral titers to below the limit of detection. Further, no S was detectable in the infected cells or supernatant (Figure 4C,D). Interestingly, in contrast to previous studies, we observed no cleavage at the MERS-CoV S S2′ site by furin during expression in HeLa cells, nor during the infection of Calu-3 cells (Figure 2C and Figure 4D) [40,75].

Here, when TMPRSS2 and furin inhibitors were used together, an additive effect was observed, indicating that TMPRSS2 was not able to fully compensate for furin in viral activation during MERS-CoV infection (Figure 4C). This result was in contrast to our initial observations that recombinant TMPRSS2 cleaved the FRET substrate representing the S1/S2 cleavage site with even higher efficiency than the S2′ motif (Figure 2B) and that MERS-CoV S was processed at both sites when co-expressed with TMPRSS2 in the absence of active furin (Figure 2C). The discrepancy in results between our co-expression system and authentic infection might result from differences in the compartmentalization of the protease and S. During transient co-expression, TMPRSS2 and S are both transported to the plasma membrane via the secretory pathway and TMPRSS2 probably cleaves the S1/S2 site during trafficking through the trans-Golgi network, in a manner similar to that established for IAV HA [77]. During infection, however, S evades the secretory pathway, as CoVs bud into the ERGIC [63]. Even though TMPRSS2 is present in this compartment, S cleavage by TMPRSS2 might be impaired. It is possible that the cleavage site is not accessible for TMPRSS2 when S is already incorporated into the viral membrane, or, alternatively, that a co-factor required for efficient S cleavage by TMPRSS2 is unavailable. We recently found that ACE2 not only interacts with TMPRSS2 but enhances the catalytic activity of TMPRSS2 in a non-catalytic manner [57]. However, little is known about ACE2 expression and its potential interaction with TMPRSS2 in the ERGIC. In the absence of furin, the TMPRSS2-mediated cleavage of S on the cell surface, at the stage of virion entry, may be sufficient to enable MERS-CoV replication, albeit at a lower efficiency than that typically observed when furin is present and performs the cleavage of S during viral egress.

The HA of H1N1/1918 has been recognized as an important virulence factor for the virus [65]. Previous studies demonstrated that it can be cleaved by co-expressed TMPRSS2 and TMPRSS4 in vitro and that both proteases facilitate HA-mediated pseudovirus entry [67,68]. Here, we found that the cleavage of the 1918 HA can also be carried out by HAT and mTMPRSS13, as has been shown for more recent H1 variants (Figure 5B) [52,58]. Viral replication and HA cleavage in infected Calu-3 human airway cells, however, were exceptionally dependent on the presence of catalytically active TMPRSS2, suggesting that cleavage by other TTSPs plays only a minor role, if any, during infection (Figure 5C,D).

Galloway et al. investigated the HA cleavage of H1-H14 and H16 by TMPRSS2 upon co-expression in vitro. Interestingly, they found that H13 but not H12 was activated by co-expressed TMPRSS2 [78]. Accordingly, we observed that H12-mediated viral replication was only slightly reduced in the absence of catalytically active TMPRSS2 while the virus displaying H13 was completely dependent on the protease (Figure 5E). In addition, our data show that the replication of an H17-bearing recombinant IAV in Calu-3 human airway cells was strikingly independent of TMPRSS2 (Figure 5E), even though it was previously demonstrated in vitro that H17 can be cleaved and activated by human TMPRSS2 [79,80]. H12 and H17 have also been shown to be cleaved by HAT [78,80], but it is unlikely that HAT compensates for TMPRSS2 in our setting as it is expressed at very low levels in Calu-3 cells [23]. All three HAs harbour a monobasic cleavage site, yet the sensitivity of the HAs to different host proteases could be due to the specific amino acid sequence or carbohydrate side chains flanking the cleavage site [81,82,83].

The sequences of H17N10 were the first to be isolated from bats [84]. H17 differs from the HAs of classical IAVs by its receptor specificity for MHC-II instead of sialic acid, and an alternative pH-independent entry into the host cell has been discussed [80,85,86]. Our data indicate that it additionally distinguishes itself from other monobasic HAs by its ability to employ other not yet identified proteases for activation. Interestingly, Freidl et al. found elevated antibody titers against H12 in *Eidolon helvum* bats in Ghana [87], prompting us to speculate that TMPRSS2-independent replication might be a common feature among bat-associated IAVs. Further studies on bat IAV H18N11 and the H9N2 sublineage recently discovered in bats, as well as investigations on HA cleavage in bat cells, are needed to elucidate the HA activation mechanism and the proteases involved in bat IAVs.

TMPRSS2 from chicken, duck, swine, and non-human primates has been shown to support the proteolytic activation of IAV HA in vitro [24,88,89,90], and most avian HA can be activated by human TMPRSS2 [24], indicating that no adaptation to specific proteases is necessary for the transmission of IAVs to new host species. However, the TMPRSS2-independent replication of bat-derived H17 and bat-associated H12 suggests that glycoprotein cleavage may play a role in transmission from or adaptation to bats. Importantly, the role of glycoprotein cleavage in inter-species transmission has been discussed in previous CoV studies. The S of MERS-CoV strains isolated from dromedaries in West Africa, where the virus is ubiquitous in camels but not transmitted to humans, has been found to be less efficiently activated by TMPRSS2 than in strains from Saudi Arabia, where zoonotic transmission is observed regularly [91,92]. The host restriction of several MERS-like bat-derived CoVs was also found to be based on proteolytic activation by TMPRSS2 [93]. MERS-CoV has been circulating for over ten years, regularly causing human disease and occasionally exhibiting human-to-human transmission. We observed a much more pronounced TMPRSS2 dependency and S cleavability for MERS-CoV than for SARS-CoV. The above studies together suggest that cleavability by TMPRSS2 is associated with enhanced transmission to, and viral replication in, humans. Moreover, the pandemic H1N1/1918, which spread very efficiently among the human population, was found to be especially dependent on TMPRSS2 cleavage in human airway cells. Recently, the emerging SARS-CoV-2 variants BA.1 and BA.2 were shown to be less efficiently cleaved by TMPRSS2 than previous variants [94,95,96]. BA.1 and BA.2 did not circulate for a long period of time, soon being overcome by BA.5, which displayed a more TMPRSS2-dependent phenotype similar to previous variants [97]. In summary, previous studies, together with the results presented in this study, suggest that the shift in protease usage to TMPRSS2 may enhance the transmission and infectivity of CoVs and IAVs in the human host.

## Figures and Tables

**Table 1:** **Role of TMPRSS2 in the activation of IAV, IBV and zoonotic CoV in Calu-3 human airway cells.** The results of this study are highlighted in bold. Additional results were described in our previous publications [23,24,31].

TMPRSS2-Dependent	TMPRSS2 Involved	TMPRSS2-Independent
H1 [23]	H9 (R-S-S-R↓) [24]	IBV [23,24]
**H1/1918**	H16 [24]	**H12**
H2 [24]		**H17**
H3 [23,24]		
H4 [24]		
H5 [24]		
H6 [24]		
H7 [23]		
H8 [24]		
H9 (R↓) [24]		
H10 [24]		
H11 [24]		
**H13**		
H14 [24]		
H15 [24]		
SARS-CoV-2 [31]	**SARS-CoV**	
**MERS-CoV**		

## Data Availability

All data are available on reasonable request.
